# Methodological barriers to studying the association between the economic crisis and suicide in Spain

**DOI:** 10.1186/s12889-017-4702-0

**Published:** 2017-09-06

**Authors:** Javier Alvarez-Galvez, Jose A. Salinas-Perez, María Luisa Rodero-Cosano, Luis Salvador-Carulla

**Affiliations:** 1 Department of Applied Health Research, University College London, 1-19 Torrington Place, London, WC1E 7HB UK; 2 Department of Quantitative Methods, Universidad Loyola Andalucía, Campus Sevilla, C/ Energía Solar, 1, Sevilla, 41014 Spain; 3 Department of Quantitative Methods, Universidad Loyola Andalucía, Campus Córdoba, C/ Escritor Castilla Aguayo, 4, Córdoba, 14004 Spain; 4 Centre for Mental Health Research, Research School of Population Health, ANU College of Health & Medicine, Australian National University, 3 Eggleston Road, Acton, Canberra, 2601 ACT Australia

**Keywords:** Great recession, Economic crisis, Suicides, Interrupted time series, Spain

## Abstract

**Background:**

The hypothetical relationship between economic recession and the increase in suicides in Spain is subject to various arguments. In addition to the inherent complexity of capturing and explaining the underlining mechanisms that could describe this causal link, different points of contention have been be identified. The period of this association and its possible starting points, the socioeconomic determinants that may explain the variation in suicide rate, and the data sources available are the main focus of controversy. The present study aims to identify the phases of association between different periods of economic recession and suicide rates, and compare the effect of different social determinants of health that have been mentioned in previous studies.

**Methods:**

We have used interrupted time series analyses to assess the impact of economic recession on national rates of suicide mortality provided by the Spanish Statistical Office (1980–2014). In an attempt to consider the factors that have affected the study of suicide in Spain, different data sources/periods, predictors, and regions in Spain were analysed.

**Results:**

The analysis revealed a positive and significant relationship between the Great Recession and suicide rates during the second period of economic recession (2011–2014), while appeared to decrease during the first recession period. However, the first decreasing trend was not statistically significant in the global analysis of the evolution of monthly suicide rates for the entire country. Both unemployment and per capita GDP were positively related to suicide trends. Finally, the regional analysis demonstrates a similar pattern in different Spanish areas.

**Conclusion:**

Although previous studies have mentioned the double-dip in the suicide rate associated with the corresponding period of double recession, our study only identify a positive relationship during the second recession period. These results points out that the major impact of economic problems might have had a delayed effect due to initial protection policies.

## Background

Suicide is a major public health problem in contemporary societies [1, 2]. In recent years, the degree of this problem has concurrently increased in relation to the global economic crisis [3]. Recent macroeconomic downturns linked to the 2008 financial crisis have been found to be associated with increasing suicide rates in Europe and in the United States [3–6]. In the European context, Spain was severely affected by unemployment and austerity policies promoted by the Troika during the period of economic crisis (i.e., the European Central Bank, International Monetary Fund and the European Commission) [7, 8]. Some studies on Spain, or specific regions such as Andalusia and Catalonia, have recently identified a positive correlation between the current economic crisis and suicides, stating that suicides had an upward trend some years after the beginning of the crisis [3, 9–14]. However, the hypothetical relationship between economic recession and an increase in suicides in Spain is subject to various arguments. In addition to the inherent complexity of capturing and explaining the underlining mechanisms that could describe this causal link, three points of contention can be identified: (1) determination of the period of this association and its possible starting points [9, 15]; (2) determination of the potentially related socioeconomic determinants to the variation in suicide rates [16]; and finally (3) determination of the appropriate data sources required to implement this research design [17].

The first point of debate is related to the identification of the relationship between the period of economic crisis and the increase in suicide rates [18, 19]. The economic downturn began in the United States during the second semester of 2007, while in Europe, the symptoms of economic crisis arrived later, mostly during the year 2008. In Spain, some studies have identified a positive relationship between the macroeconomic downturn and the rate of suicide from the year 2008 [9]. However, this relationship has been recently questioned since it is hard to postulate that the economic recession, which was an event that occurred suddenly in March 2008 [15], had a direct impact on the rate of suicide. In fact, some studies posit that the major impact of economic problems might have had a delayed effect due to initial protection policies [20, 21]. A second area of dispute is related to the possible macroeconomic determinants of increasing suicide rates. In general, unemployment is the most widely used indicator of a financial crisis [10, 11, 22], especially in Mediterranean countries such as Spain [23]. This measure has been proven to have either inconsistent or contradictory results [16] since there are two-way relationships that mask these effects, such as the existing between unemployment and mental illness [24]. Finally, we have to address the methodological problem of combining different data sources to study the relationship between economic downturns and suicide rates. Suicide data provided by the Spanish Statistical Office (Instituto Nacional de Estadística [INE]) differs considerably from data identified by forensic pathologists, which adds more complexity to the analysis and makes it problematic to obtain a clear interpretation of this phenomenon, especially when neither of the two data sources have been found to be any more reliable than the other [17].

Consistent with these problems, the present study aims to (1) identify phases of association between different periods of economic recession and suicide rates; (2) compare and assess the effect of different social determinants of health that have been mentioned in specialised literature; and (3) avoid the methodological problem of using different data sources and time periods by performing an interrupted time series analysis.

Relative to these objectives, our hypotheses are as follows:

- H1: There is a positive relationship between the financial crisis and the rate of suicide in Spain that started during the second period of recession (2011–2014), which is a moment that coincides with a period of economic cuts and with the end of previous rescue packages;
- H2: Unemployment will demonstrate a positive association with suicide rates, while increasing GDP and social expenditure should produce an inverse association with suicide rates.

## Methods

### Data and variables

In this study, we are using monthly data on the rate of suicide as provided by the INE. Because INE changed the system through which suicides were recorded [25], this trend is composed of two main data series: (1) monthly suicide data from legal records from 1980 to 2006; (2) monthly suicide data by cause of death from 2007 to 2014. As in other countries, in Spain the suicide data are different according to the official register that collects them, so the both data series are not directly comparable [17, 26]. The study variables are the crude suicide rates per 100,000 population for total, male and female population. Both sources do not provide data by age group hampering the age-standardization of rates. Regarding the possible variations introduced by sex, the Spanish yearly sex ratio was steady in this time span with values within the range [0.96–0.98]. Thus, the differences are small and enable the analysis of the crude rates directly.

**Fig. 1:**
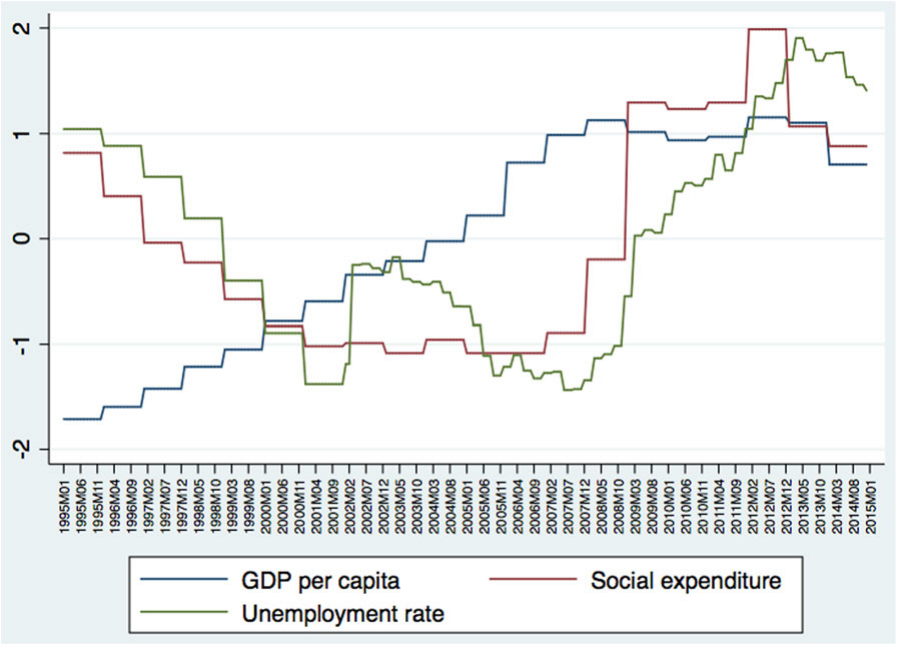
Trends in GDP per capita, unemployment rate and social expenditure during the period of analysis

According to previous studies, different variables have been included in the analysis to test the relationship between the socioeconomic factors and suicide. The variables under study are as follows: (1) per capita gross domestic product (GDP) to study the relationship between the economic stature of individuals and the rate of suicide; (2) unemployment rate as a predictor that has been widely understood to be a cause of mental illness and suicide; and (3) social expenditure to study the possible effect of variations in the size of the welfare state in Spain (Table 1). Figure 1 describes variation in these predictors during the period under analysis (values have been standardized for comparative purposes). The main effects will be analysed separately in the models and finally compared to assess their joint impact on suicide mortality.

**Table 1:** Descriptive statistics of variables in the analysis and multicollinearity tests

Variable	Observations	Mean	Std. Dev.	Min	Max	VIF	1/VIF
Monthly suicide rates	240	0.54	0.11	0.24	0.80		
GDP per capita	240	25,591.65	5670.31	15,888.00	32,140.00	1.43	0.70
Social expenditure (% GDP)	240	41.72	3.16	38.30	48.00	4.17	0.24
Unemployment rate	240	17.62	5.07	10.35	27.26	3.74	0.27

### Interrupted time series analysis

In an attempt to consider the factors that have affected the study of suicide in Spain, an interrupted time series analysis (ITSA) with Stata 14.0 [27] was conducted to analyse different data sources and contextual circumstances associated with the economic crisis in Spain.

Because we were using only one group for the analysis (i.e., monthly suicide rate per total population), the standard interrupted time series regression model is described as follows [28–31]:

$$ {Y}_t={\beta}_0+{\beta}_1{T}_t+{\beta}_2{X}_t+{\beta}_3{X}_t{T}_t+{\epsilon}_t $$

where *Y*_*t*_ is the outcome variable measured at each equally spaced *t* (time), *T*_*t*_ is the time elapsed from the beginning of the study, *X*_*t*_ is a binary variable representing the intervention (i.e., periods of pre-intervention = 0, all others = 1), and finally, the interaction term in the equation, which is represented by *X*_*t*_ *T*_*t*_. In this single-group model, *β*_*0*_ is the intercept of the outcome variable, *β*_*1*_ is the slope of the outcome variable prior to the introduction of the intervention (i.e., change or interruption in the time series), *β*_*2*_ represents the change in the level of the outcome that occurs in the period immediately following the intervention, and *β*_*3*_ is the difference between pre- and post-intervention slopes of the outcome. Therefore, the aim of the analysis is to look for significant *p*-values in either *β*_*2*_ to identify any possible and immediate treatment effect, or in *β*_*3*_ to identify a treatment effect over time [29, 30]. Thus, the ITSA was used to conduct two different but complementary analysis.

First, the evolution of the monthly suicide rate per 100,000 inhabitants for the entire country was analysed together with the socioeconomic factors. The suicide dataset was divided in three periods. The first period, ranged from January 1995 to December 2006, coincides exactly with the period where suicide was collected by legal records. In the second period, the evolution of suicide was analysed from January 2007 until July 2011, which was the period of occurrence of the first economic recession. Finally, the third period from August 2011 to December 2014 was analysed, which coincides with the second economic recession, a change in government, and the full establishment of the Troika policies for economic budget cuts. Unlike previous analyses, the advantage of our model was that it allowed for the study of these contextual changes (i.e., variations in measurement data and/or historical events in data series that could contribute to the explanation and interpretation of the results).

Second, to increase the validity of the initial results, the analysis was stratified by gender and compared to different provinces in Spain. These regions were chosen according to two basic criteria: population density and geographical distribution. Therefore, we selected the most populated regions distributed along the Spanish territory, to (1) avoid the effects of variation associated with a lower sample size in the least populated provinces, while (2) considering the possible variability that was associated with different regions among different autonomous communities. The selected provinces were as follows: Asturias (Asturias), Biscay (Basque Country), Barcelona (Catalonia), Madrid (Community of Madrid), Pontevedra (Galicia), Seville (Andalusia), Toledo (Castile-La Mancha), and Valencia (Valencian Community). In this second study, we used the data of yearly suicide rate by sex from 1980 to 2014. The effect of a previous period of recession was added (i.e., the economic recession of 1992–1993) with the objective of increasing the understanding of the relationship between economic downturns and suicide. In this model, the effect of other predictors such as GDP, social expenditures and unemployment was not included to work with the main effect of time and the intervention periods with a lower number of observations (i.e., 35 years of follow-up).

## Results

**Fig. 2:**
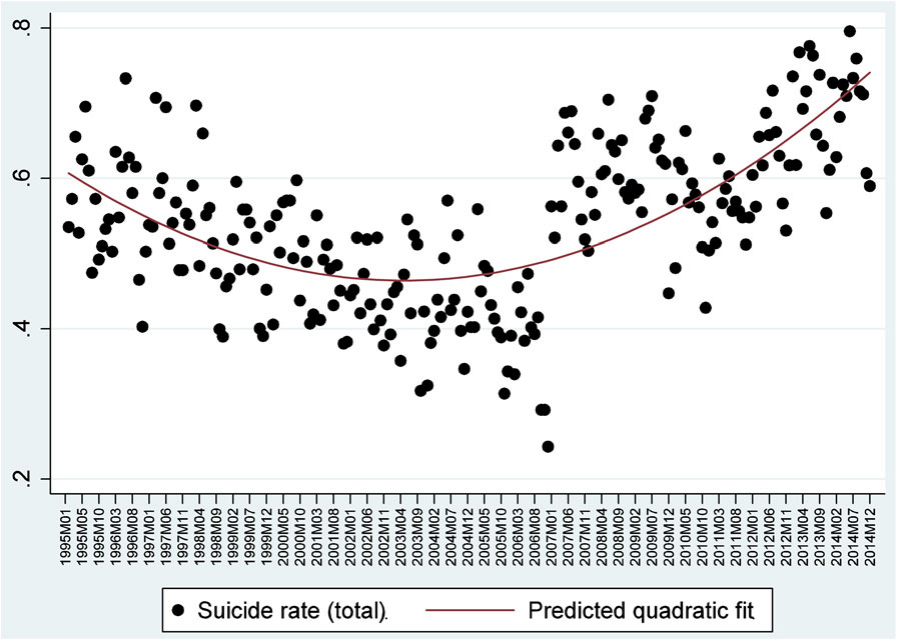
Monthly suicide rate per 100.000 inhabitants in Spain (1995–2014)

Suicides have evolved following an increasingly statistically significant trajectory (Fig. 2). Since 1995, the suicide rate experienced a progressive decline until 2006, when the rate shifted and began to increase significantly, experiencing a sharp increase from the year 2007 until its peak in 2014 (linear fit *R* ^*2*^ = 0.35, *p* < .001; *R* ^*2*^ quadratic fit = 0.58, *p* < .001). However, this trend of monthly suicide rates in Spain may encourage misinterpretations. As explained previously, two different suicide data series are combined in this analysis, which must be considered. Despite the inference that there was an increase in suicides during the 2007–2014 period, which could hypothetically be linked to the economic downturn, this incremental rise was related to a change within the registry in terms of changing the database of suicides from legal records (1995–2006) to suicides by cause of death (2007–2014). In fact, the economic crisis would not actually hit Spain until 2008.

### Monthly evolution of suicides and socioeconomic factors

**Fig. 3:**
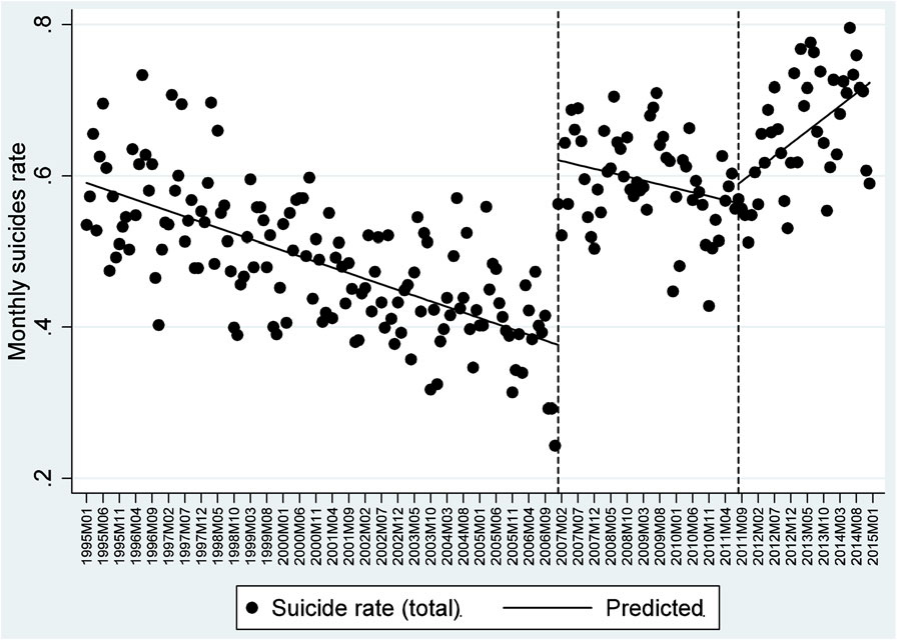
Monthly suicide rate per 100.000 inhabitants in Spain through ITSA (1995–2014)

The results of the analysis showed that during the first data series (1995–2007), the suicide rate was significantly reduced (Fig. 3). During the period in which the first economic downturn occurred (2007–2011), the analysis does not show a statistically significant relationship for that trend, despite an adjustment line that shows a downward trend. Therefore, because we had the second series of INE data (i.e., suicides classified by cause of death), it would not be possible to attribute any increase in suicide to the first period of the economic recession. This means that the suicide rate remained relatively stable during the initial period (i.e., 2007 to 2011), irrespective of the first economic recession. However, during the second period of economic downturn, which concurred with the context that economic cuts were generalised all along the Spanish public sector, a statistically significant increase in the suicide rate was present. This finding can be clearly observed in Table 2 where the effect of the two periods of recession are compared (2007–2011 vs. 2011–2014). From this viewpoint, the results show a turning point that coincides with one of the most critical moments of the economic crisis, with the period of the recession when many of the social benefits of previous policies were either removed or reduced.

**Table 2:** ITSA including the effects of GDP per capita, social spending and the unemployment (1995–2014)

*Variables*	*Model 1*	*Model 2*	*Model 3*	*Model 4*
Initial time	−0.002 (<0.001)^***^	−0.001 (<0.001)^***^	−0.001 (<0.001)^***^	−1.118 (0.378) ^***^
Intervention1 (2007)	0.204 (0.001)^***^	−0.241 (0.025) ^***^	0.248 (0.023)^***^	100.337 (14.365) ^***^
IntervXtime1 (2007)	0.002 (0.031)^**^	<0.001 (0.001)	−0.001 (0.001)	0.688 (0.686)
Intervention2 (2011)	0.004 (0.010)	0.026 (0.028)	0.024 (0.028)	−1.346 (12.359)
IntervXtime2 (2011)	0.005 (0.027)^***^	0.005 (0.001) ^***^	0.005 (0.001) ^***^	2.042 (0.716) ^***^
GDP	0.001 (0.001)^**^			0.007 (0.003) ^**^
Social expenditure		0.001 (0.005)		−1.545 (2.479)
Unemployment rate			0.003 (0.002)^*^	1.964 (1.057)^*^
Constant	0.372 (0.113)^**^	0.518 (0.120) ^**^	0.530 (0.048)^***^	143.342 (96.300)
*Recession periods*	*Model 1*	*Model 2*	*Model 3*	*Model 4*
T2007–2011	−0.001 (0.001)	−0.001 (0.001)	−0.002 (0.357)^**^	−0.430 (0.428)
T2011–2014	0.004 (0.001)^***^	0.004 (0.001)^***^	0.003 (0.001)^***^	1.6120 (0.555)^***^
Stat. F	50.10^***^	52.72^***^	54.88^***^	64.88^***^
Obs.	240	240	240	240

*p* ≤ 0.05^*^, *p* ≤ 0.01^**^, *p* ≤ 0.001^***^

As observed in the regression model (Table 2), the initial level of the rate of monthly suicide was estimated at 0.372, and this rate seemed to significantly decrease every month (−0.002, *p* < 0.001). Afterwards, in the first year of the intervention (2007), a significant increase in the rate of monthly suicide was observed (0.204, *p* < 0.001), which was related to the change in the database and was followed by a significant increase in this trend (0.002, *p* < 0.001) when compared to the pre-intervention period. The second intervention (2011) was not statistically significant due to the lower differences in the previous trend, but these differences became statistically significant during the period between 2011 and 2014 (0.005, *p* < 0.001). On the other hand, in an attempt to analyse the factors that could be related to the evolution of the monthly rate of suicide during these different periods, the effect of three predictors commonly mentioned in recent literature was described: [1] per capita gross domestic product (model 1); [2] social expenditures, which were measured as a percentage of GDP (model 2); and [3] unemployment rate (model 3). The results demonstrated a statistically significant effect both for the GDP and for the unemployment rate, while social spending did not present as a significant relationship. In addition, the model 4 included simultaneously the effect of the three predictors and the results support these findings. The only difference is that social expenditures had a negative relationship with suicide rate, though it was not significant.

A comparison of the two intervention periods is presented (2008–2011 recession vs. 2011–2014 recession) in the lower portion of Table 2. From this comparison, we may observe that the monthly rate of suicide appeared to decrease during the first economic downturn, but this trend of decline was only statistically significant in the third model when the effect of unemployment was included (0.002, *p* < 0.001). However, during the second period of recession, the rate of suicide increased between 0.003 and 0.004 every month, which showed fully significant growth across the three models.

### Comparison across Spanish provinces

The period prior to the 1992 recession did not demonstrate a clear association to the suicide rate (Table 3). During this initial period, the relationship between the economic downturn and the rate of suicide was only positive for females in Madrid and Biscay. Barcelona would have demonstrated a similar trend if a shorter period was considered (e.g., 1992–1999), but the longer period from 1992 until 2007 resulted in a negative adjustment line. The suicide rate in females seemed to decrease in Biscay, Madrid, Toledo and Valencia; however, the suicide rate increased for males in Asturias, Barcelona and Valencia during 2008–2011, which was the first period of the Great Recession. The second period of recession (2011–2014) affected males in Asturias, Barcelona, Biscay, Madrid, Pontevedra, Seville, and Toledo but also affected females in Madrid, Pontevedra, Seville and Valencia. Finally, statistically significant differences in the trends in the suicide rate between males and females were identified in Barcelona, Madrid, Pontevedra and Seville during this period. These differences are clear if we observe the global tendency for the country, which means that during the period between 2011 and 2014, the rate of suicide in the male population was 0.424 points higher than the rate of suicide in the female population (*p* < 0.001).

**Table 3:** Comparing the effect of economic recessions on yearly suicide trends by gender between the provinces and Spain

	*Asturias*			*Barcelona*			*Biscay*		
Recessions	1992	2008	2011	1992	2008	2011	1992	2008	2011
Male	−0.232^**^	2.947^***^	1.199^***^	−0.116	0.068	0.560^***^	0.122	−1.282	0.508^***^
Female	−0.087	0.452	0.193	−0.085^**^	0.054^***^	−0.020	0.110^**^	−0.100^***^	0.414
Difference	−0.145	2.496^***^	0.192	−0.031	0.259^***^	0.580^***^	0.011	−0.182	0.095
	*Madrid*			*Pontevedra*			*Seville*		
Recessions	1992	2008	2011	1992	2008	2011	1992	2008	2011
Male	0.139	−0.797^***^	1.847^***^	−0.110	−1.678^*^	1.734^***^	−0.90	−0.621	1.391^***^
Female	0.064^*^	−0.176^***^	0.855^***^	0.060^**^	−0.609	0.605^***^	−0.003	−0.180	0.149^***^
Difference	0.075	−0.622^***^	0.991^***^	−0.170	−1.069	1.128^***^	−0.087	−0.441	1.249^***^
	*Toledo*			*Valencia*			*Spain*		
Recessions	1992	2008	2011	1992	2008	2011	1992	2008	2011
Male	0.187	0.243	2.032^***^	−0.105^***^	0.631^***^	0.107	−0.016	−0.543^***^	0.783^***^
Female	−0.041	−0.455^***^	0.018	−0.036^*^	−0.123^***^	0.244^*^	−0.013	−0.210^***^	0.359^***^
Difference	0.228	0.698	2.015	−0.069^*^	0.754^***^	−0.137	−0.003	−0.334^***^	0.424^***^

*p* ≤ 0.05^*^, *p* ≤ 0.01^**^, *p* ≤ 0.001^***^

**Fig. 4:**
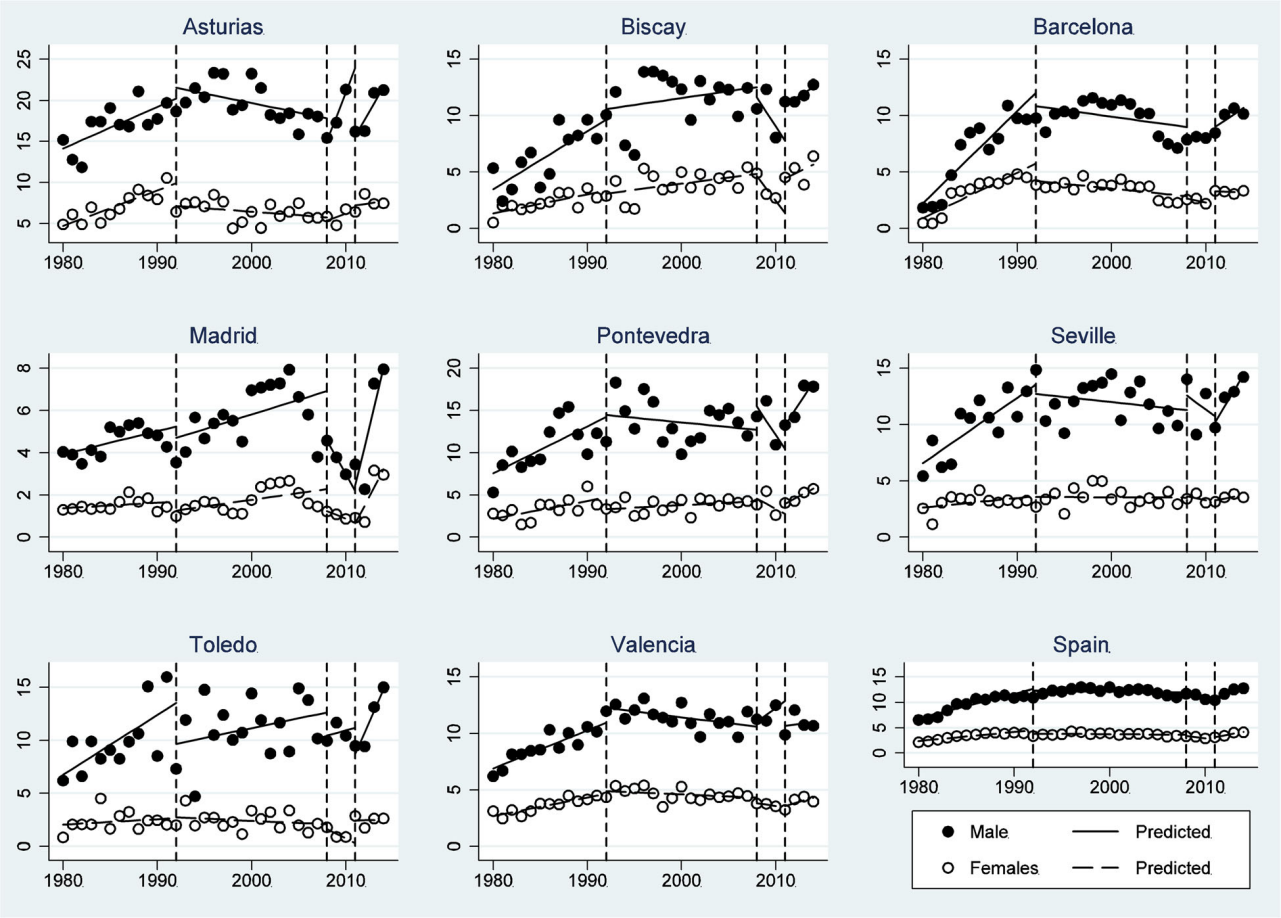
Comparison of yearly suicide rates per 100.000 inhabitants in Spanish provinces by gender: Asturias, Biscay, Barcelona, Madrid, Pontevedra, Seville, Toledo and Valencia

It is interesting to observe (Fig. 4) that the pattern of suicide is similar in some geographic regions. Indeed, a similar trend was found in Mediterranean regions (Barcelona and Valencia), central regions (Madrid and Toledo), and in northern regions (Biscay and Pontevedra). On the other hand, it is relevant to consider how trends in the rate of suicide are more stable for women than for men, whose tendency suffered higher variations over the period analysed.

## Discussion

The present study aimed to (1) identify the relationship between the different periods of economic recession and the rate of suicide; (2) describe and evaluate the effects of different social determinants of health mentioned in the literature on suicide; and (3) avoid the methodological problem of using different data series and periods by performing an interrupted time series analysis. In relation to the first objective, the first hypothesis was that the positive relationship between the financial crisis and suicide rates occurred during the second period of the recession from 2011 to 2014. Besides, the second hypothesis, linked to the second objective, was that unemployment had a positive influence while GDP per capita and social expenditure had negative effect on the suicide rates.

Based on the first hypothesis, the analysis revealed a positive and significant relationship between the Great Recession and the rate of suicide during the second period of economic recession (2011–2014), while the rate of suicide appeared to decrease during the first period (2007–2011). However, the first decreasing trend was not statistically significant in the global analysis of the evolution of monthly suicide rates for the entire country, but only for the analysis by time period (per year). On the other hand, it is fundamental to understand that this relationship will vary depending on the next series of available data; therefore, the clear and positive effect during the period from 2011 to 2014 could be moderated by subsequent suicidality. In the short term, the analysed trends seem to demonstrate that during the last years of economic crisis, the Spanish population has lost economic wealth but has also lost the buffer of previous social policies, and the combination of this double-loss might be related to increasing suicide rates in this country. Obviously, we will need long-term follow-up on this trend if we want to improve the accuracy of our predictions and the reliability of these measurements.

The effect of the second recession on the suicides has been fewer studied in the literature. Previous studies, which have been able to access more recent data, have mentioned the double-dip in the suicide rate associated with the corresponding period of double recession [10, 13]. However, that trend is not clear according to our analysis. In particular, we have observed that the rate of suicide was also higher in 2007, which was not exclusive to 2008, and we have to consider that delayed effect [20, 21]. Despite the plausibility that during the first year of the economic downturn, a certain proportion of individuals may have committed suicide because of their economic problems, the loss of socioeconomic status is not immediate since there were public and social protection policies, such as an universal access to education, health care, social services and social transfers, that could have reduced the impact of economic problems. However, the decreasing effect during the period from 2008 to 2011 might also be related to the resilience of the population during the first years of the economic downturn [32], which was a protective social effect that could have been eroded during the progression and accumulation of socioeconomic difficulties and mental health conditions. Although this is just a conceivable hypothesis, we will need additional qualitative information to study this assumption in depth.

About the second hypothesis, both unemployment and per capita GDP were positively related to suicide trends. The indirect relationship between economic problems and suicide is well-known because these circumstances might push people to mental illness, which has also been found to have an association with suicide [3–6, 33, 34]. It is necessary to note that the unemployment benefits in Spain may have skewed the effect, since the impact of unemployment in people’s life could be less dramatic than in other countries where there are no social benefits [14, 35]. Unemployment also could affect the number of suicides indirectly, since it is associated to mental disorders that may trigger suicide ideation [36, 37]. Beside this, the positive effect of per capita GDP in this context may be initially confusing. As explained above, we have to consider the delayed effect between socioeconomics changes and suicide [20, 21], which may have influenced this relationship. Thus, the economic upturn may take a time to affect the suicide rate as had occurred with the downturn previously.

Moreover, it is not possible to infer from our findings that at individual level a higher socioeconomic status was related with greater predisposition for committing suicide since we have used a macroeconomics indicator, the risk of suicide could be linked to people who stayed outside of the slow macroeconomic improvement. Another possible explanation is that the effect that the labour reform carried out in Spain during this period has had on the loss of the socioeconomic status of the population [7, 8]. Due to this, the socioeconomic status of the population has deteriorated to individual level, despite there is a global economic improvement. This situation may lead to people with a higher socioeconomic status, which have been influenced by processes of social mobility, may be more susceptible to suicide. In fact, social drifts have been found to be related to mental health disorders and suicide in modern societies [36–38] in which an individual exposed to this process can lose not only their previous economic status, but also their social capital (i.e., relationships, social influence, trust, etc.). Therefore, the individual relationship between socioeconomic status and suicide risk in Spain needs to be further researched at case level.

Contrary to expectations, social expenditure did not show a statistically significant association with suicide trends, which is likely due to social spending has shown an irregular trend because the different policies applied by subsequent governments. However, future analyses should explore this relationship according to the specific figures of social expenditure (e.g., health, family benefits, pensions, etc.).

When comparing different regions (Fig. 4), we checked that the northern zone (Pontevedra and Asturias) is the region with the highest suicide rate, especially Asturias. Followed by the south area (Seville) and the central zone (Toledo). On the contrary, it can be observed that the regions with the highest employment (Madrid and Barcelona) have the lowest rate of suicide. Both the similarity in the pattern of suicide found in some geographical areas (Mediterranean, central y northern regions), and the most stable trends in the rate of suicide for women than for men coincide with results of previous research [9, 10, 18, 39].

Additionally, we observed that provinces such as Madrid demonstrated a large variability in the trends of suicide, and it was likely that this higher variation might have been related to the deficient data registry or even to population dynamics. In the case of Madrid, an update on the suicide data was made available from the Anatomic Forensic Institute in the years 2013 and 2014, which is probably the explanation for the association with higher peaks for this region in those years [17]. Therefore, it is relevant to understand that we are always going to find methodological and statistical problems that are associated with the frequency and reliability in the data registry when working with the rates of suicide [17, 26]. Consequently, it could be hazardous to provide a definite response regarding future trends in suicide since there exists a wide-spread debate about the quality of the Spanish suicide dataset in the academic field, which is likely the result of the change in the official registration of this social phenomenon.

Despite the advantages of the method of study, the research has a number of limitations that should be mentioned. First, age and sex may have great effect on the suicide rates of geographical areas or time spans with different demographic structure (5,12). Therefore, standardization of rates is necessary to carry out accurate comparisons [40] but has been impossible of performing the age-standardization of the suicide rates in this study due to unavailability of data. However, the sex-standardization was not necessary because of the minor variation in the sex ratio during the time span in Spain, and the use the suicide rates by sex in the regional study. Second, other limitation is under-reporting of suicide deaths, which is a debated issue [41], but it is not possible to avoid because the research is based on official statistics. Third, it is totally plausible that cumulative effects mental health degradation associated to the first recession could have increased suicide mortality in the second period, however, we do not have data on mental health to confirm this hypothesis. Finally, ecological fallacy is a common concern in studies which use aggregate data as this research [42]. The interpretation of the relationships between socioeconomic factors and suicide rates, which have been found at state scale, should not be inferred directly for the individual scale or the conclusions may be biased.

## Conclusion

The easy and fast response to if the Great Recession is associated with increasing rates of suicide in Spain according to the present analysis is affirmative but only partially. The Great Recession has been found to be positively related to the increasing suicide rate, but this indirect relationship was observed exclusively during the second period of recession from 2011 and 2014. In other words, the economic crisis has possibly affected the rate of suicide during the period characterised by economic cuts in social protection.

With regard to the effects of different social determinants, we can affirm that both unemployment and per capita GDP were positively related to suicide trends in Spain at state level. The positive effect of per capita GDP should be further studied since may have affected both to people who were marginalized of the macroeconomic improvement and to people who were influenced by processes of social mobility. In addition, social expenditure did not show a statistically significant association with suicide trends what may have been due to the different orientation of the social benefits carried out in Spain by the successive governments.

In addition, the study carried out by regions shows similar trends to previous research, which allows us to confirm that the methodology used was correct and adequate to analyse a complex problem such as suicide. Even so, this is the first phase of the study so it will be necessary a more detailed analysis that confirms the results and allows us to better detect the causes of this problem.

Finally, despite the limitations associated with the use of suicide data, our approach presents some advantages compared with previous works. The main benefit of our approach is that it allows for the study of contextual changes associated with different time periods. In other words, our analysis measured the variations in the magnitude of historical events associated with a series of data, and unlike other studies, this approach can increase the understanding and interpretation of the results across different time periods and contextual circumstances. Finally, according to recommendations from the World Health Organization [1], this predictive model – adjusted to available data series – may also help national strategies focused on suicide prevention through the implementation of analytical solutions that might avoid methodological problems associated with research on suicide.
